# Effect of Si Content on Microstructures and Electrochemical Properties of Al-xSi-3.5Fe Coating Alloy

**DOI:** 10.3390/ma16237407

**Published:** 2023-11-28

**Authors:** Yufeng Wu, Ying Shen, Qi Wang, Yuhang Liu, Dongming Shi, Ya Liu, Xuping Su

**Affiliations:** 1 School of Materials Science and Engineering, Changzhou University, Changzhou 213164, Chinashenying_youxiang@163.com (Y.S.); m15370809989@163.com (Y.L.); yliu@cczu.edu.cn (Y.L.); 2 Jiangsu Key Laboratory of Materials Surface Science and Technology, Changzhou University, Changzhou 213164, China

**Keywords:** microstructure, electrochemical performance, Al-xSi-3.5Fe coating alloy

## Abstract

Hot-dip aluminum alloy is widely used in the engineering fields. However, during the aluminum plating process, Fe inevitably enters and reaches a saturation state, which has a significant impact on the corrosion resistance and microstructure of the coating. Currently, adding Si during the hot-dip aluminum process can effectively improve the quality of the coating and inhibit the Fe-Al reaction. To understand the effect of Si content on the microstructure and electrochemical performance of Al-xSi-3.5Fe coating alloys, the microstructure and post-corrosion morphology of the alloys were analyzed using SEM (Scanning Electron Microscope) and XRD (X-ray Diffraction). Through electrochemical tests and complete immersion corrosion experiments, the corrosion resistance of the coating alloys in 3.5 wt.% NaCl was tested and analyzed. The results show that the Al-3.5Fe coating alloy mainly comprises α-Al, Al_3_Fe, and Al_6_Fe. With the increase in Si addition, the iron-rich phase changes from Al_3_Fe and Al_6_Fe to Al_8_Fe_2_Si. When the Si content reaches 4 wt.%, the iron-rich phase is Al_9_Fe_2_Si_2_, and the excess Si forms the eutectic Si phase with the aluminum matrix. Through SKPFM (Scanning Kelvin Probe Force Microscopy) testing, it was determined that the electrode potentials of the alloy phases Al_3_Fe, Al_6_Fe, Al_8_Fe_2_Si, Al_9_Fe_2_Si_2_, and eutectic Si phase were higher than that of α-Al, acting as cathode phases to the micro-galvanic cell with the aluminum matrix, and the corrosion form of alloys was mainly galvanic corrosion. With the addition of silicon, the electrode potential of the alloy increased first and then decreased, and the corrosion resistance results were synchronous with it. When the Si content is 10 wt.%, the alloy has the lowest electrode potential and the highest electrochemical activity.

## 1. Introduction

Corrosion is one of the primary forms of metal component failure, and corrosion problems exist in various fields of engineering construction. Metal corrosion not only causes enormous economic losses but also leads to catastrophic accidents. Developing anti-corrosion technology is urgently needed to reduce corrosion losses and promote resource conservation. Therefore, seeking methods and technologies to prevent steel corrosion is significant. Among them, surface engineering technology for surface modification is one of the most active frontier fields in materials science, which is a crucial method to prolong the service life of steel and improve economic benefits [1,2,3].

Hot-dip galvanizing is the most important anticorrosion method for steel, which is widely used in automobile, ship, bridge, pressure vessel and other industries, but the shortage of zinc resources is becoming more and more severe. It is an inevitable trend and requirement to develop new materials for the hot plating of steel that can replace zinc, and aluminum is the most promising material to replace zinc [4]. Hot-dip aluminizing is a simple and efficient surface-coating technology [5]. The hot-dip aluminizing coating has a metallic luster surface and excellent high-temperature oxidation resistance, good corrosion resistance, wear resistance, and reflectivity to light and heat [6,7,8]. During the continuous hot-dip aluminizing process, iron and aluminum atoms diffuse and react with each other, thus, forming a layer of the Fe-Al intermetallic compound layer. After cooling, a hot-dip aluminizing layer is formed on the surface of the steel matrix, which can effectively improve the corrosion resistance of steel [9]. The intermetallic compound layer mainly comprises an inner layer of Fe_2_Al_5_ and an outer layer of FeAl_3_. The corrosion resistance and adhesion of the hot-dip coating largely depend on the properties and morphology of the inner layer of Fe_2_Al_5_ and the outer layer of FeAl_3_. However, there are still many problems in theory, performance, and preparation technology, which limit the wide application of continuous hot aluminum plating. The thickness of the Fe-Al intermetallic compound layer in the coating increases rapidly. It has greater brittleness and lower fracture strength, which deteriorates the bonding strength of the interface between the steel matrix and the aluminum-based coating and reduces the machinability of hot-dip aluminized steel [10,11,12,13].

As an additive element in hot-dip aluminizing, silicon can not only improve the fluidity of the aluminum liquid, reduce the temperature of the aluminum liquid, and reduce the oxide impurity content in the aluminum liquid but also significantly inhibit the increase in the thickness of the Fe/Al reaction layer. The main reason is that Si can inhibit the rapid growth of the η-Fe_2_Al_5_ phase [14,15,16]. Springer et al. [17] studied the reaction between low-carbon steel and pure aluminum and between low-carbon steel and a Al-5 wt.% Si alloy and concluded that the total thickness of the reaction layer mainly depends on the parabolic growth controlled by the diffusion of the η phase (Fe_2_Al_5_), which exhibits orientation-dependent growth kinetics. When 6 wt.% Si is added, the thickness of the alloy layer can be effectively reduced. Some scholars believe that Si atoms can fill the atomic vacancies in Fe_2_Al_5_, preventing aluminum atoms from preferential fast diffusion along the c-axis direction [18,19,20]. Our research group believes that silicon changes the diffusion channel of coating growth and then changes the coating phase composition, thereby reducing the growth rate of the alloy.

During the continuous hot-dip plating process, the steel plate and the sinking roller system are corroded by the aluminum liquid, and the Fe element inevitably dissolves into the aluminum liquid. Therefore, after a production period, the iron in the aluminum bath can reach the saturation point. The higher iron content in the Al-Si-Fe bath leads to the formation of several intermetallic phases, such as hexagonal τ_5_ (α-Al_8_Fe_2_Si), monoclinic τ_6_ (β-Al_9_Fe_2_Si_2_), τ_11_ (δ-Al_4_Fe_2_Si), as well as other binary Al-Fe compounds (Al_3_Fe, Al_5_Fe_2_, etc.) [21,22,23]. Li et al. studied the effect of Fe on the mechanical properties of aluminum alloys and found that the appearance of an Al-Fe-Si phase decreased the plasticity and strength of the alloy, but the high-temperature mechanical properties increased [24]. Piotr’s research found that the separation and reduction in iron-rich phases may play a role in the removal of Fe from Al-Si alloys [25]. Kakinuma et al.measured the open-circuit potentials of the bulk intermetallic compounds and Al-matrix of AA1050 without intermetallic particles. The cathodic reactivity on bulk Al-Fe was higher than that on bulk Al-Fe-Si under as-polished condition [26]. The rich iron content in the aluminum matrix greatly influences the coating’s corrosion resistance and electrochemical properties.

Although studies on Al-Fe-Si alloys have been reported, they have mainly focused on the influence of trace iron content on the alloy structure. There have been no reports on the effects of Si content variation on the microstructure and electrochemical properties of aluminum coating alloys in the iron-saturated state. This article conducted qualitative research on five types of iron-saturated aluminum alloys and conducted electrochemical analysis and microstructure characterization of the coating alloys to explore the impact of changes in Si content on the structure and electrochemical properties of Al-Fe-Si alloys.

## 2. Experiment

### 2.1. Material Preparation

The experimental alloys in Table 1 were melted in the XK-25Z medium frequency induction furnace (Lanhui Technology, Xi’an, China) at 720 °C. The required raw materials, aluminum particles (99.99 wt.%, Licheng Innovation Metal Materials Technology, Beijing, China), iron particles (99.99 wt.%, Licheng Innovation Metal Materials Technology, Beijing, China), and aluminum-silicon master alloy (Al-12.24 wt.%Si, Licheng Innovation Metal Materials Technology, Beijing, China) were placed in a drying oven (Yiheng Scientific Instrument, Shanghai, China) and dried for 1 h to remove surface moisture. After pure aluminum was melted, the Si element was added as an intermediate alloy, and the Fe element was added as high-purity iron particles wrapped in aluminum foil. The alloys were stirred thoroughly after adding the alloying elements, and hexachloroethane (Sigma-Aldrich, Shanghai, China) was used for refining and degassing. The alloys were kept at 720 °C for 24 h. Finally, the liquid alloys were cast into a metal mold preheated to 350 °C.

S. Pontevichi [27] found that the solubility of iron in the liquid aluminum is approximately 3.5 wt.% at 727 °C. This was combined with the melting temperature of this experiment, and it was determined that the Fe content in the coating alloy studied in this article is 3.5 wt.%.

After cooling, the alloys were cut into 10 mm × 10 mm × 5 mm alloy blocks using wire cutting (Sanguang Technology, Suzhou, China) and ground step by step on 400-mesh to 2000-mesh silicon carbide sandpaper (Sanguang Technology, Suzhou, China). After polishing with a diamond polishing agent (Maifeng Metering Technology, Taizhou, China), the samples were cleaned with absolute ethanol (Najing Reagent, Nanjing, China) and blown dry with cold air for later use.

### 2.2. Microstructure Analysis

A JSM-6510 scanning electron microscope (Oxford Instrument, Oxford, UK) and an Oxford energy dispersive spectrometer (EDS, Oxford Instrument, Oxford, UK) were used with an accelerating voltage of 20 kV to observe the surface morphology of the sample before and after corrosion and analyze the composition and distribution of the relevant elements on the alloy surface and cross-section.

The XRD measurements were performed on a Siemens D500 X-ray diffraction system (Siemens, Munich, Germany) with a scanning speed of 2°/min and 2θ ranging from 10° to 90° to analyze the phase composition of the alloy. MDI Jade6 software (Jade6.0, 2017, Materials Data, New City Square, PA, USA) was used to perform background subtraction analysis on the XRD spectrum and compared with the International Center for Diffraction Data (ICDD) PDF card.

### 2.3. Electrochemical Analysis

The PARSTAT-4000A electrochemical workstation (AMETEK, Philadelphia, PA, USA) was used to test the open circuit potential (OCP), electrochemical impedance spectroscopy (EIS), and potentiodynamic polarization curve (Tafel) of the sample with a surface area of 1 cm^2^. A three-electrode system was used, in which the sample was the working electrode, the reference electrode was a saturated calomel electrode (KCl), and the auxiliary electrode was a platinum electrode. The electrochemical test was carried out under a constant-temperature water bath at 25 °C.

When electrochemical testing was performed, the sample was first immersed in 3.5 wt.% NaCl solution (Sigma-Aldrich, Shanghai, China) for 3600 s to measure the open circuit potential in the steady state. Dynamic potential polarization and electrochemical impedance spectroscopy (EIS) tests were performed using an electrochemical potentiostat (AMETEK, Philadelphia, PA, USA). In the EIS test, a sinusoidal voltage with a frequency range of 10^5^–10^−2^ Hz and an amplitude of 5 mV was used to measure the EIS at the OCP and use ZSimpWin software (1.0 of ZSimpWin, 2017, AMETEK, Philadelphia, PA, USA) to fit the EIS. The electrochemical dynamic potential polarization scanning interval was ±0.5 V (vs. OCP), and the scanning speed was 1 mV/s. CView2 software (2.0 of CView, 2007, AMETEK, Philadelphia, PA, USA) was used to fit and analyze the polarization curve. The scanning Kelvin probe force microscope (SKPFM) experiments were carried out on electrochemical samples using an atomic force microscope (Bruker Multimode, Salbuluken, Germany).

### 2.4. Full Immersion Corrosion Experiment

The size of the corrosion immersion sample was 10 × 10 mm^2^. The samples before and after soaking were dried and weighed using a precision balance (Huazhi, Putian, China). The sample was soaked in the 3.5 wt.% NaCl solution at 25 °C for 720 h. The samples were then washed with absolute ethanol and dried immediately. First, a scanning electron microscope (SEM) was used to observe the corrosion surface morphology of the sample. Then, the corroded samples were cut in the vertical direction, ground, and polished, and the cross-sections of the corroded samples were observed using SEM.

To elucidate the effect of alloy element Si content on corrosion products and alloy electrochemical properties in a 3.5 wt.% NaCl environment, the soaked alloy samples were dried in a drying oven for 24 h, and their corrosion products and relative contents were tested using XPS: X-ray Photoelectron Spectroscopy (Siemens, Munich, Germany).

## 3. Results

### 3.1. Microstructure and Phase Analysis of the Alloy

#### 3.1.1. Al-Fe-Si Ternary Phase Diagram

**Figure 1:**
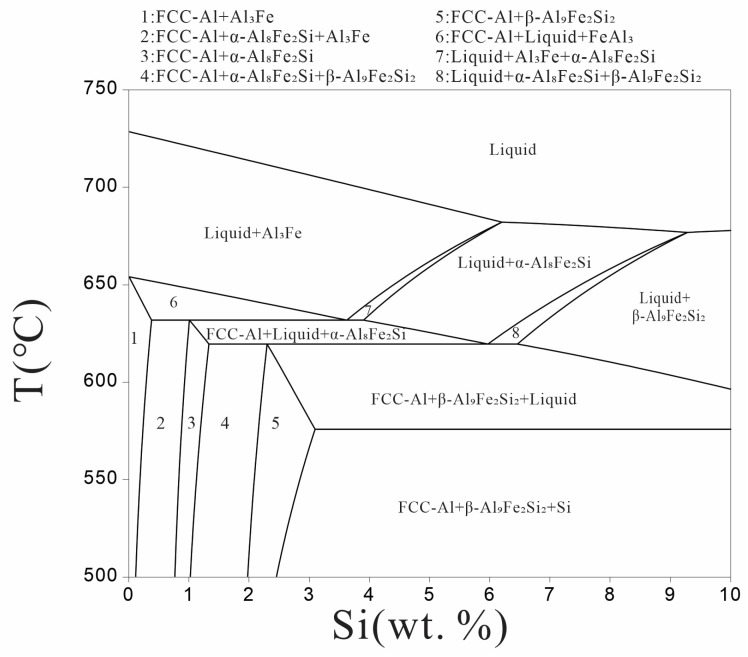
Vertical Cross-Section Phase Diagram of Al-xSi-3.5Fe Alloy.

The vertical section phase diagram of the Al-xSi-3.5Fe coating alloy was calculated with cost50B database in Pandat software (2022 of Pandat, 2022, CompuTherm, Middleton, WI, USA), as shown in Figure 1. As the temperature gradually cooled from 720 °C, the initial phases forming in the Al-3.5Fe alloy were Al_3_Fe, according to the phase diagram. The final solidified microstructure of the alloy consists of FCC (Face-centered cubic)-Al and Al_3_Fe phases. As the addition of Si in the alloy increased to 1 wt.%, the τ_5_(α-Al_8_Fe_2_Si) phase occurred during cooling. The Al_3_Fe phase disappeared in the Al-2Si-3.5Fe alloy at room temperature, and the iron-rich phases were composed of τ_5_(α-Al_8_Fe_2_Si) and τ_6_(β-Al_9_Fe_2_Si_2_) phases. As the Si content was above 2 wt.%, the τ_5_(α-Al_8_Fe_2_Si) phase disappeared, and the eutectic Si phase gradually precipitated at 577 °C. When the Si content was above 4 wt.%, the solidified Microstructure(microstrucyure) of Al-xSi-3.5Fe coating alloy was always composed of FCC-Al, Si, and τ_6_(β-Al_9_Fe_2_Si_2_) phases.

#### 3.1.2. Microstructure and Morphology of Alloy before Corrosion

**Figure 2:**
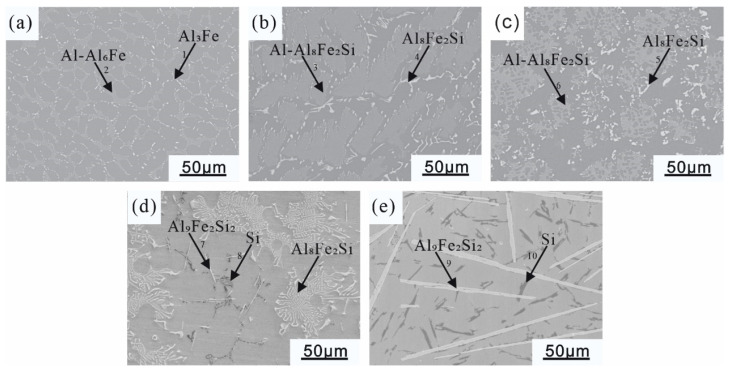
SEM (Scanning Electron Microscope) morphology of Al-xSi-3.5Fe coating alloy: (**a**) Al-3.5Fe; (**b**) Al-1.0Si-3.5Fe; (**c**) Al-2.0Si-3.5Fe; (**d**) Al-4.0Si-3.5Fe; (**e**) Al-10Si-3.5Fe.

**Figure 3:**
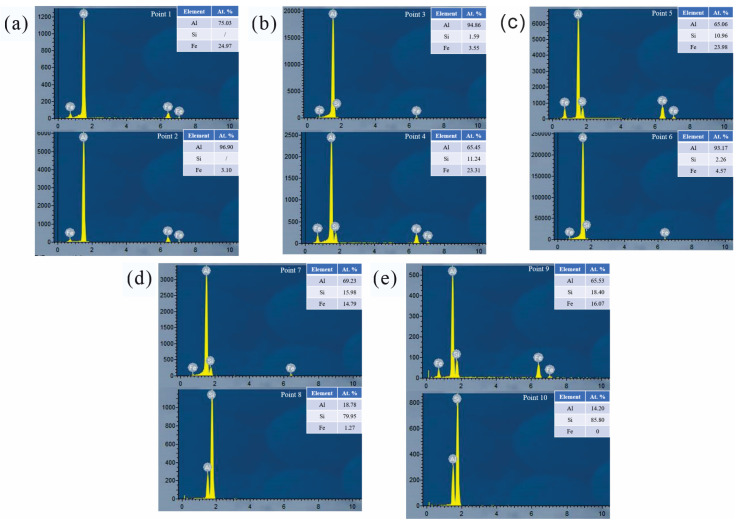
Corresponding EDS results for each point in Figure 2. (**a**) Points 1 and 2 in Figure 2a; (**b**) Points 3 and 4 in Figure 2b; (**c**) Points 5 and 6 in Figure 2c; (**d**) Points 7 and 8 in Figure 2d; (**e**) Points 9 and 10 in Figure 2e.

**Figure 4:**
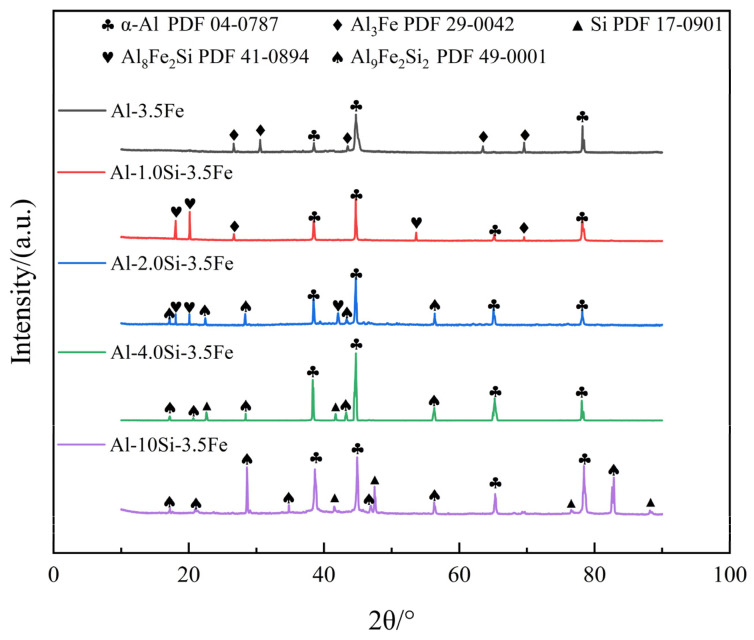
X-ray diffraction pattern of the Al-xSi-3.5Fe coating alloy.

Combining the SEM morphologies (Figure 2), EDS results (Figure 3), and X-ray diffraction patterns (Figure 4) of the coating alloys with different Si contents, the phases in the alloys were determined. The original Al-Fe alloy without adding Si consisted of three phases: the gray α-Al matrix phase, the white fine primary Al_3_Fe phase, and the dispersed Al-Al_6_Fe eutectic phases, which were evenly distributed [28]. As shown in Figure 2b,c, with the addition of the Si element, the Al-Fe phase disappeared in the Al-1Si-3.5Fe alloy, and the iron-rich phase transformed into a Chinese character-shaped τ_5_(α-Al_8_Fe_2_Si) phase and a dispersed Al-τ_5_ eutectic phase. The dispersed Al-τ_5_ eutectic phase decreased, and the iron-rich τ_5_(α-Al_8_Fe_2_Si) phase increased in the Al-2Si-3.5Fe alloy. As shown in Figure 2d,e, coarse dendritic τ_6_(β-Al_9_Fe_2_Si_2_) and τ_5_ coexist in the Al-4Si-3.5Fe alloy. Excess Si reacted with the aluminum matrix to form a eutectic Si phase. τ_5_ disappeared in the Al-10Si-3.5Fe alloy, and the microstructure became coarse. The grayish-white τ_6_(β-Al_9_Fe_2_Si_2_) and black eutectic Si phases were seen [29].

### 3.2. Microstructure Analysis of Alloy after Corrosion

#### 3.2.1. Surface Morphology of Alloy after Corrosion

**Figure 5:**
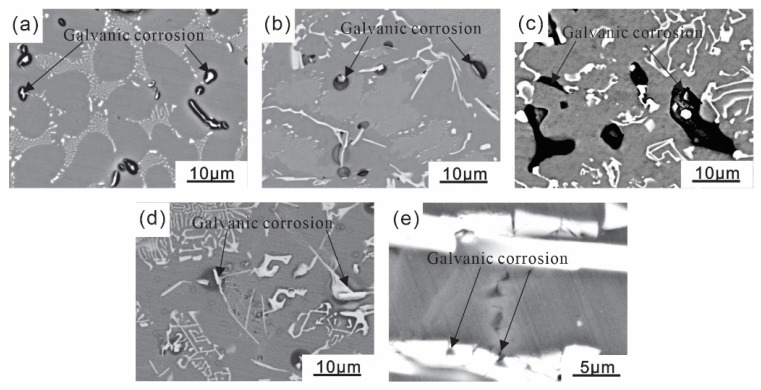
Surface corrosion morphology of Al-xSi-3.5Fe coating alloy: (**a**) Al-3.5Fe; (**b**) Al-1.0Si-3.5Fe; (**c**) Al-2.0Si-3.5Fe; (**d**) Al-4.0Si-3.5Fe; (**e**) Al-10Si-3.5Fe.

Figure 5 shows the corrosion morphology of Al-xSi-3.5Fe alloys immersed in a 3.5% NaCl solution for 30 min. After soaking, corrosion pits (black areas) appeared on the surface of the alloys, and insoluble corrosion products were present. From observing the position of the corrosion pits, it can be found that when the Si content is 0–1 wt.%, the corrosion pits of the alloys start around the Al_3_Fe and Al_8_Fe_2_Si phases, and galvanic corrosion occurs with the aluminum matrix, and the no corrosion phenomenon occurs around the dispersive phase; when the Si content is 2 wt.%, galvanic corrosion occurs between the aluminum matrix and Al_8_Fe_2_Si; when the Si content is 4 wt.%, galvanic corrosions occurred between the aluminum matrix and Al_8_Fe_2_Si, Al_9_Fe_2_Si_2_, and eutectic Si, and the corrosion pits were mainly concentrated around the eutectic Si phase; when the Si content is 10 wt.%, there were noticeable corrosion pits around the eutectic Si phase, the Al_9_Fe_2_Si_2_ phase was broken under the corrosion influence, and galvanic corrosion occurred between Al_9_Fe_2_Si_2_ and eutectic Si [30].

**Figure 6:**
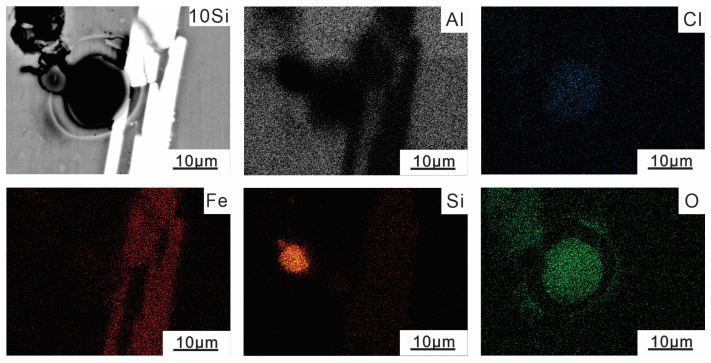
EDS distribution map of elements in Al-10Si-3.5Fe alloy after corrosion.

Figure 6 shows the SEM image and corresponding element distribution of Al-10Si-3.5Fe alloy after corrosion. Based on the enrichment of the Fe and Si elements, these phases were eutectic Si and Al_9_Fe_2_Si_2_, consistent with the results of the previous phase analysis. From the distribution of black corrosion areas, it can be determined that the corrosion pits are located in the middle of the eutectic Si phase and Al_9_Fe_2_Si_2_ phase. The eutectic Si and Al_9_Fe_2_Si_2_ formed a galvanic cell with the aluminum matrix, resulting in galvanic corrosion. The content of O and Cl elements in the corrosion area was very high, indicating the deposition of corrosion products such as hydroxide and chloride on the surface of the alloy.

#### 3.2.2. Cross-Sectional Morphology of Alloy after Corrosion

**Figure 7:**
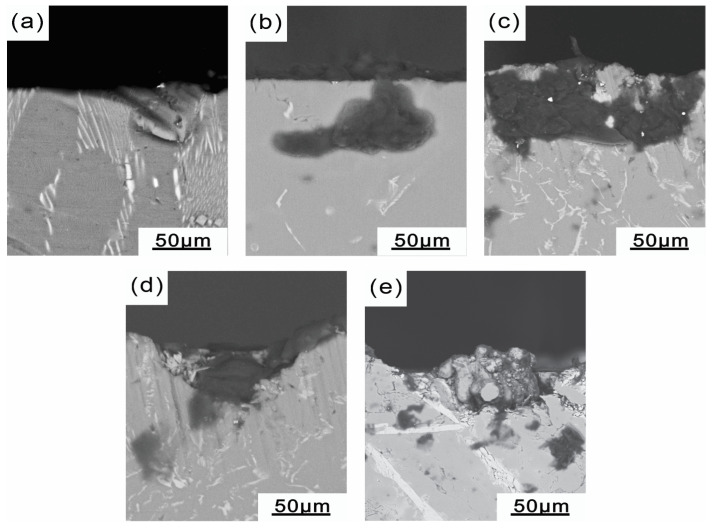
Corrosion cross-sectional morphology of the Al-xSi-3.5Fe coating alloy: (**a**) Al-3.5Fe; (**b**) Al-1.0Si-3.5Fe; (**c**) Al-2.0Si-3.5Fe; (**d**) Al-4.0Si-3.5Fe; (**e**) Al-10Si-3.5Fe.

In the cross-sectional morphology of the Al-xSi-3.5Fe coating alloy (Figure 7), corrosion pits were found around the iron-rich phase and eutectic silicon phase. Figure 7a displays noticeable corrosion pits and corrosion stripes on the cross-section of the Al-3.5Fe alloy, indicating the poor corrosion resistance of the alloy. Figure 7b–d show that the corroded morphology of the alloys with silicon addition becomes more uniform, and the corrosion pits and corrosion stripes were mainly around Al_8_Fe_2_Si, Al_9_Fe_2_Si_2_, and the eutectic silicon phase. Figure 7e indicates that when the silicon content was too high, while the Al_9_Fe_2_Si_2_ phase formed with the Fe element, the excess Si included the eutectic Si phase, which acted as the cathode phase and accelerated the corrosion rate of the alloy.

#### 3.2.3. Corrosion Products

**Figure 8:**
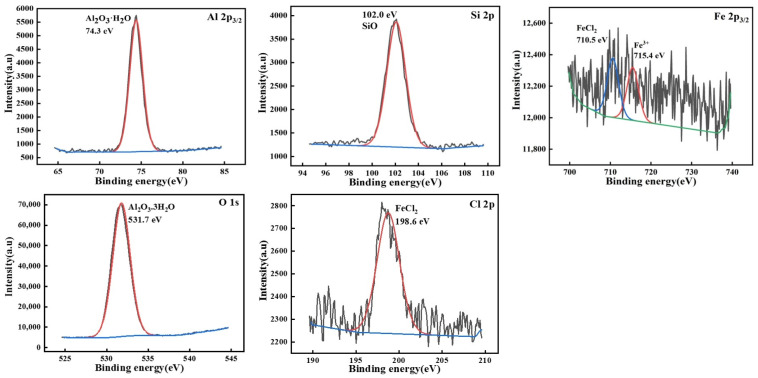
High-resolution X-ray photoelectron spectroscopy of corrosion products of Al-10Si-3.5Fe alloy. The black line represents the raw data, the red line represents the fitted data and the blue line represents the background.

To further investigate the relationship between the corrosion products of Al-10Si-3.5Fe alloy and the sacrificial anode performance, the compounds formed on the surface of the alloy samples soaked for 720 h were characterized by X-ray photoelectron spectroscopy (XPS), as shown in Figure 8. The chemical states of the Al and Fe alloy elements in XPS confirmed the presence of corrosion products, corresponding to the results in Figure 6. It was confirmed by the XPS database (NIST) that the Al 2p3/2 spectrum mainly consists of the Al^3+^ valence state, and only the Al_2_O_3_∙H_2_O peak existed. The O 1s spectrum was the OH^−^ peak, corresponding to the corrosion products of hydroxide, with Al(OH)_3_ accounting for the highest proportion. The Fe 2p3/2 spectrum of alloy corrosion products had peaks at 710.5 eV and 715.4 eV, corresponding to Fe^2+^ and Fe^3+^, respectively. The Cl^−^ peak at approximately 198.6 eV corresponds to Cl 2p3/2, which reacted with Fe^2+^ to form the corrosion product FeCl_2_.

### 3.3. Electrochemical Test and Analysis

**Figure 9:**
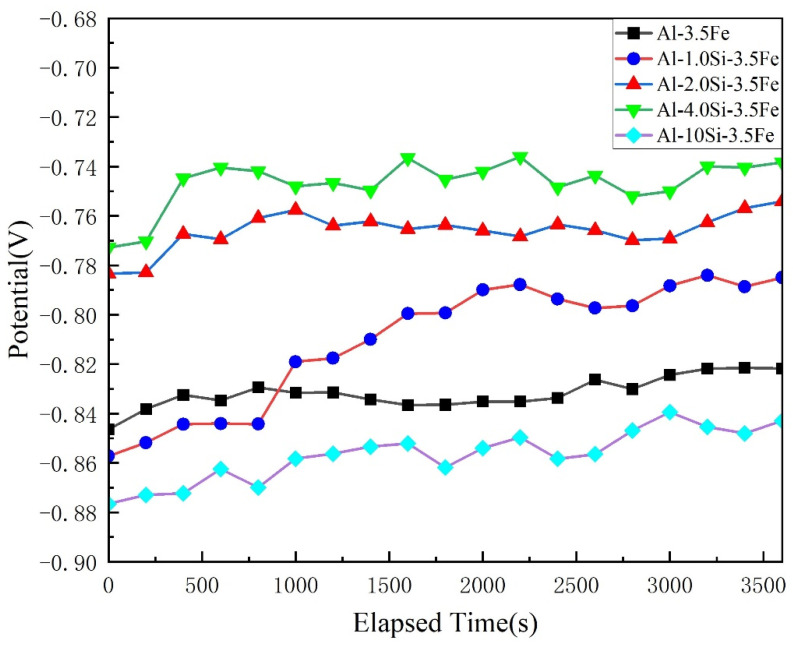
Open circuit potential of Al-xSi-3.5Fe coating alloy.

**Figure 10:**
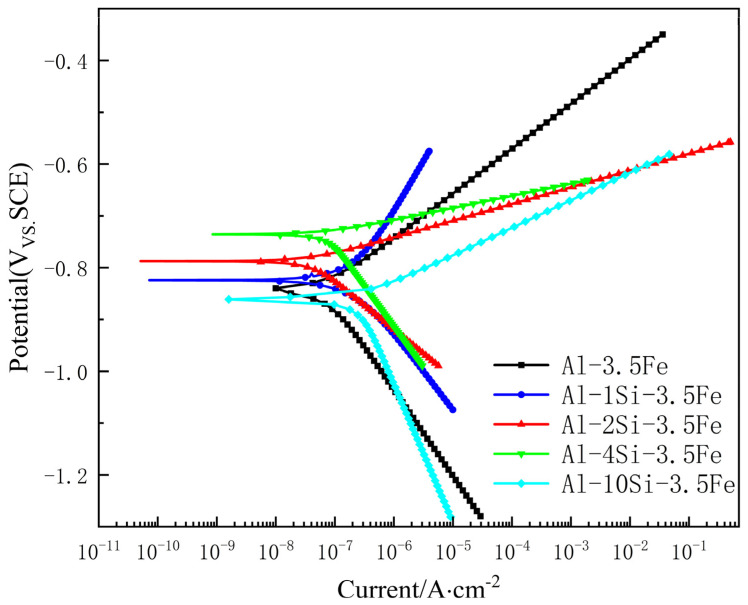
Potentiodynamic polarization curve of Al-xSi-3.5Fe coating alloy.

**Figure 11:**
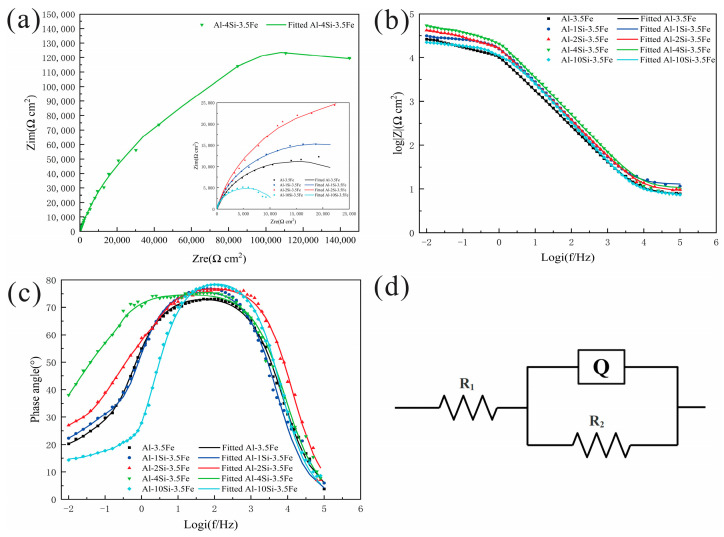
Electrochemical impedance spectra of Al-xSi-3.5Fe coating alloy: (**a**) Nyquist plot; (**b**) Bode plot; (**c**) Bode Phase plot; (**d**) equivalent circuit diagram.

**Figure 12:**
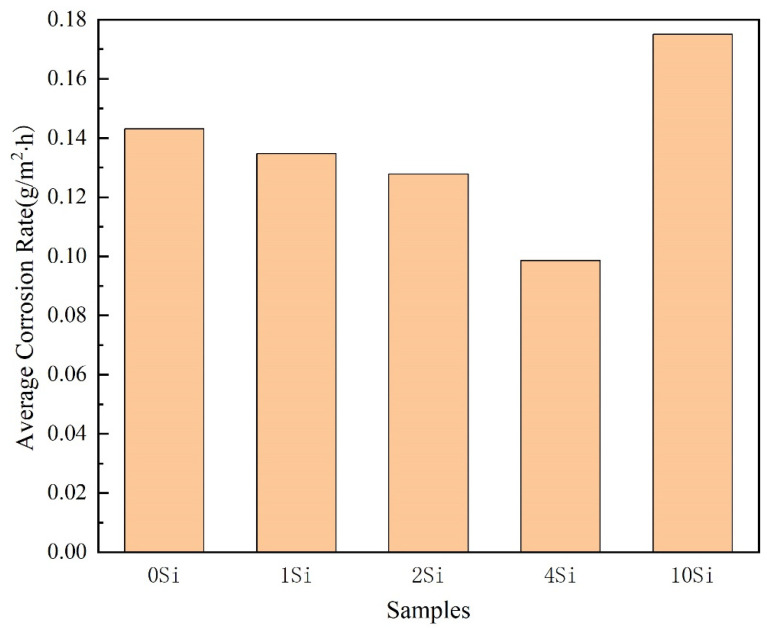
Corrosion weight loss bar chart of Al-xSi-3.5Fe coating alloy.

To study the effect of Si content on the electrochemical behavior of the Al-xSi-3.5Fe coating alloy, electrochemical corrosion tests were carried out on alloys with different compositions at 25 °C. The electrochemical behaviors are shown in Figure 9, Figure 10, Figure 11 and Figure 12.

#### 3.3.1. Open Circuit Potential

Figure 9 shows the open circuit potential (OCP) of Al-Si-Fe alloys with different Si contents. The samples were immersed in 3.5 wt.% NaCl aqueous solution and OCP was tested for 3600 s. It can be seen from Figure 9 that the OCP of Al-3.5Fe alloy without the Si element was relatively stable with minor fluctuations, and the potential was −0.82 V. The OCP of Al-1Si-3.5Fe alloy became active and tended to be steady after the 2500 s. By comparing the steady-state OCP, it was found that with the increase in Si content, the potential of the Al-xSi-3.5Fe alloy shifted positively and then negatively. As the Si content reached 4 wt.%, the electrode potential of the alloy rose to the highest value (−0.73 V). The electrode potential increased by nearly 100 mV. The electrode potential of the Al-10Si-3.5Fe alloy dropped to the lowest value (−0.85 V); the maximum electrode potential difference of the alloy reached 120 mV, but the fluctuation range was small.

#### 3.3.2. Polarization Curve

It can be seen from Figure 10 that with the increase in Si content, the corrosion potential of the alloy first rose and then fell, and the corrosion current density first rose and then fell. When Si content was 4%, the corrosion potential of the alloy was the highest at −0.741 V, which is close to the previous research results [31,32], and the self-corrosion current density was the lowest.

The key electrochemical parameters were obtained by tangentially fitting the cathode and anode regions of the polarization curve, as shown in Table 2. Among them, E_corr_ is the self-corrosion potential of the corrosion system, I_corr_ is the self-corrosion current density of the corrosion system, B_a_ is the anodic Tafel constant, and B_c_ is the cathodic Tafel constant. The experimental results show that the addition of the Si element has a specific reducing effect on the anodic polarization rate, reduces the oxidation reaction of aluminum, enhances the anodic dissolution activity, and improves the discharge efficiency. The B_c_ values of aluminum alloy sacrificial anode samples with different Si contents are all greater than B_a_ values, indicating that the cathodic hydrogen evolution reaction of the aluminum alloy sacrificial anode in seawater is a control step and, thus, plays an influential protective role.

The average corrosion rate $V_{corr}(mm/y)$ is related to the corrosion current density $I_{corr}(A/{cm}^{2})$. It can be estimated using the following relationship [33]:

(1) $$U_{corr}=3270\times M\times I_{corr}/\rho$$

Among them, 3270 is a constant for establishing the unit of corrosion rate, $I_{corr}$ is the corrosion current density, $\rho (g\cdot {cm}^{-3})$ is the material density, and $M{(g\cdot equiv}^{-1})$ is the equivalent weight.

#### 3.3.3. Electrochemical Impedance

Figure 11a showed the EIS curves of the alloy. The Nyquist plots of the Al-xSi-3.5Fe alloy only consist of one capacitance arc. The capacitive arc diameter of the Al-10Si-3.5Fe alloy was the smallest, and that of the Al-4Si-3.5Fe alloy was the largest. The capacitive arc’s diameter gradually dropped and soared with the increased Si content. In this regard, the charge transfer resistance (Rp) of the Al-10Si-3.5Fe was approximately 11.27 kΩ·cm^2^ which is the lowest corrosion resistance among the five samples. The Rp for Al-4Si-3.5Fe was approximately 308.10 kΩ·cm^2^, which was the highest corrosion resistance [34,35]. Generally, the smaller the capacitive arc diameter, the greater the corrosion rate of the sample and the better the electrochemical activity of the alloy [36]. The Bode curves shown in Figure 11b exhibited the same characteristics. In the relationship between impedance and frequency, the impedance of the Al-10Si-3.5Fe alloy was the lowest at high frequencies, while that of Al-4Si-3.5Fe alloy was the highest. The impedance followed a trend of first declining and then peaking with the increase in Si content. Figure 11c showed the relationship between phase angle and frequency, with the increase in Si content, the phase angle in the low-frequency region first rose and then fell. The peak of the phase angle gradually moves towards the high-frequency direction, indicating that the impedance value of the anodic film first rose and then fell. The dissolution activity of the anode first declined and then soared. The Al-4Si-3.5Fe alloy has the highest phase angle in the high-frequency range, exhibiting better capacitive characteristics and a better ability to prevent corrosive ions from invading. The Al-10Si-3.5Fe alloy has the lowest phase angle, indicating better activity. Figure 11a–c suggest that the trend of changes in various alloys is consistent, meaning that the corrosion mechanism of the alloys under different composition states is the same.

To quantitatively evaluate the electrochemical corrosion behavior of the Al-10Si-3.5Fe alloy, the equivalent circuit in Figure 11d was used to fit the EIS. The equivalent circuit mainly consists of two parts. The first part is Rs, which represents the solution resistance; the second part is Rp and Q, which represent the double layer capacitance between the aluminum alloy substrate and the electrolyte solution, corresponding to the double layer capacitance and charge transfer resistance of the Faraday reaction. The charge transfer resistance Rp represents the difficulty of the substrate losing electrons. The EIS circuit fitting results are shown in Table 3. The larger the Rp, the higher the corrosion resistance of the alloy. Therefore, the Al-4Si-3.5Fe alloy has the best corrosion resistance, while the Al-10Si-3.5Fe alloy has the strongest activity.

#### 3.3.4. Alloy Full Immersion Experiment

The whole immersion experiment can shorten the experimental period and quickly obtain corrosion products and surface morphology. The A1-3.5Fe-xSi alloy was immersed in a 3.5 wt.% NaCl solution for 720 h, and the surface corrosion was evident with varying degrees of corrosion products attached. Figure 12 is a graph based on the data in Table 4. According to the chart data, the weight of samples in each group increased after 720 h of the total immersion experiment due to different degrees of galvanic corrosion on the alloy during the immersion process, and the corrosion products attached to the alloy surface did not entirely fall off.

Within the Si content range of 0–10 wt.%, the self-corrosion potential of the alloy first shifts positively and then negatively, and the alloy’s dissolution activity and corrosion rate first decrease and then increase. The corrosion rate of the A1-10Si-3.5Fe alloy is 0.1833 g/m^2^·h, and its self-corrosion potential is −0.8429 V, indicating the best dissolution activity. The 720-h full immersion test has the fastest corrosion rate, with a corrosion current density of 0.24255 μA·m^−2^. The alloy has the smallest current generated by self-corrosion and high current efficiency. The corrosion rate of the A1-4Si-3.5Fe alloy is 0.0986 g/m^2^·h, and its self-corrosion potential is −0.7383 V, indicating the best corrosion resistance and the slowest corrosion rate.

Generally, the larger the corrosion current, the faster the corrosion rate, the better the electrochemical activity, and the more corrosion products [37]. The results of this full immersion corrosion experiment are consistent with the conclusions of the previous electrochemical impedance spectroscopy, polarization curve, and fitted self-corrosion parameters.

## 4. Discussion

### 4.1. Microstructure of Coating Alloy

In this study, we employed scanning electron microscopy (SEM) and X-ray diffraction (XRD) techniques to characterize the microstructure of Al-xSi-3.5Fe alloys used for hot-dip coating. Combined with the results of Figure 2, Figure 3 and Figure 4, we observed that the changes and outcomes of various phases in the coating alloy showed some discrepancies with the calculated phase diagram in Figure 1. For hypoeutectic Al-Fe alloys under non-steady-state solidification conditions, the appearance of stable phase Al_3_Fe and metastable phase Al_6_Fe is attributed to the varying cooling rates. The study revealed that in the binary system, the non-equilibrium phase transition of AlFe intermetallic compounds occurred at temperatures below 400 °C, forming Al_6_Fe compounds. In contrast, the Al_3_Fe compounds emerged at temperatures above 400 °C [38]. When the cooling rate T_L_ is lower than 1.5 K/s, there is a coexistence region for the Al_3_Fe and Al_6_Fe phases in the eutectic mixture [28]. During the solidification process of the alloy, temperature gradients between the alloy surface and the interior result in increased cooling rates, promoting the formation of metastable phases and eutectic structures.

Furthermore, this study also investigated the relationship between the formation of compounds in Al-Si-Fe alloys and both the cooling rate and the content of Fe and Si elements. It was found that a higher Fe/Si concentration ratio and a faster cooling rate both favored the increase in τ5 (Al_8_Fe_2_Si) phase. Additionally, the addition of Si reduced the critical cooling rate. When the alloy solidified, exceeding the critical cooling rate led to the final solidification structure being composed solely of the τ5 phase. During the solidification process, if there is a significant difference between the external temperature and the temperature of the molten alloy, and if the cooling rate is too fast, high-temperature phases solidify first, while low-temperature phases do not form. This study provides important theoretical insights into understanding the solidification process of aluminum-based composite materials and optimizing their manufacturing processes [29,39].

### 4.2. Corrosion Phenomenon of Coating Alloy

Aluminum alloys exposed to the atmospheric environment form a protective oxide film on their surface. However, the existence of iron-rich phases in the intermetallic compounds can affect the thickness uniformity of the oxide film, resulting in surface defects [40]. At the same time, corrosive chloride ions in the environment can penetrate through these defects and interact with the oxide film, reducing the corrosion resistance of the alloy [41]. The alloy phases Al_6_Fe, Al_3_Fe, Al_8_Fe_2_Si, and Al_9_Fe_2_Si_2_ and the eutectic Si phase are the main intermetallic compounds in Al-xSi-Fe alloys, and their electrochemical activity is lower than that of the aluminum matrix, resulting in potential differences and local galvanic corrosion phenomena around the matrix.

**Figure 13:**
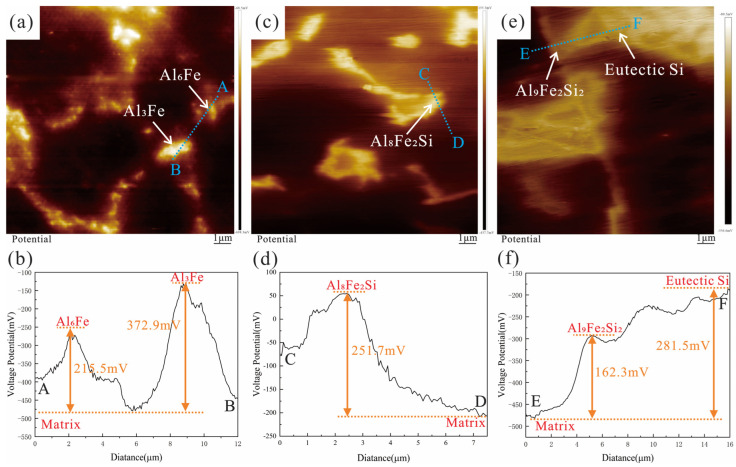
SKPFM analysis of Al-xSi-3.5Fe coating alloys after immersion in 3.5 wt.% NaCl solution: (**a**) SKPFM potential map of Al-3.5Fe; (**b**) cross-sectional analysis of SKPFM-Al_3_Fe and Al_6_Fe particles; (**c**) SKPFM potential map of Al-2.0Si-3.5Fe; (**d**) cross-sectional analysis of SKPFM-Al_8_Fe_2_Si particles; (**e**) SKPFM potential map of Al-10Si-3.5Fe; (**f**) cross-sectional analysis of SKPFM-Al_9_Fe_2_Si_2_ and Si particles.

To further investigate the influence of the second phase on the electrochemical performance of the alloy, SKPFM was used to measure the potential difference between the second phase particles and the aluminum matrix [42]. As shown in Figure 13, Al_6_Fe, Al_3_Fe, Al_8_Fe_2_Si, Al_9_Fe_2_Si_2_, and eutectic Si have higher electrode potentials than the aluminum matrix. The potential differences between Al_3_Fe and Al_6_Fe relative to the matrix were +372 mV and +215 mV, respectively (Figure 13b); the potential difference between Al_8_Fe_2_Si and the matrix was +251 mV (Figure 13d); and the potential differences between Al_9_Fe_2_Si_2_ and Si relative to the matrix were +162 mV and +281 mV, respectively (Figure 13f). The electrode potentials of the iron-rich phase and eutectic Si were more positive relative to the matrix α-Al, resulting in potential differences and micro-galvanic cell formation, causing galvanic corrosion around the second phase and generating corrosion points.

For aluminum alloys, the initial development of corrosion is often related to the metal interphase compounds present in the alloy. Combined with the corrosion morphology of the alloy shown in Figure 9, when the alloy does not contain Si elements, the corrosion is mainly due to the significant potential difference between the Al_3_Fe phase and the aluminum matrix, resulting in galvanic corrosion. The surrounding dispersoid Al_6_Fe phase has a high Al content and a relatively small potential difference compared to the aluminum matrix, and no corrosion occurs; when the Si content is 1–2 wt.%, the Al_8_Fe_2_Si phase undergoes galvanic corrosion with the aluminum matrix. The Fe and Si contents in Al-Al_8_Fe_2_Si phases are relatively low, and there is no significant corrosion due to the slight potential difference compared to the aluminum matrix; when the Si content is 4 wt.%, galvanic corrosion occurs between the eutectic Si phase and the aluminum matrix due to the larger potential difference, while the potential difference of the iron-rich phase is relatively small; when the Si content is 10 wt.%, the eutectic Si phase increases significantly, and its electrode potential is higher. There is a potential difference between the aluminum matrix and Al_9_Fe_2_Si_2_, causing galvanic corrosion of the aluminum matrix and the Al_9_Fe_2_Si_2_ phase.

**Figure 14:**
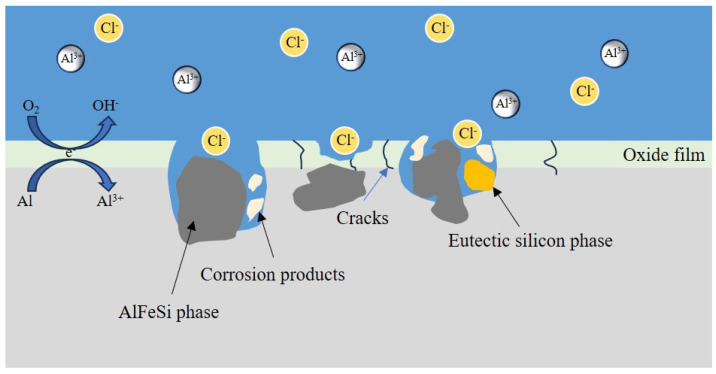
Schematic diagram of the local corrosion of the Al-10Si-3.5Fe sample.

The corrosion pits preferentially initiate at the locations of the iron-rich second phase particles and eutectic Si phase [43]. The locally cathodic iron-rich and eutectic Si phases enhance the cathodic reaction, leading to galvanic corrosion near the second phase. As a result, the α-Al matrix preferentially dissolves near the second-phase particles. Figure 14 describes the corrosion mechanism of the Al-10Si-3.5Fe coating alloy. The alloy is exposed to an environment rich in corrosive ions (Cl^−^), penetrating the aluminum matrix through the defect points around the iron-rich and eutectic Si phases, dissolving the oxide film on the surface, and causing corrosion. The iron-rich phase AlFeSi, AlFe particles, and eutectic Si phase act as cathodic phases, forming a potential difference with the adjacent aluminum matrix and generating many micro-galvanic cells, resulting in galvanic corrosion.

### 4.3. Electrochemical Analysis of Coating Alloys

The electrochemical behavior of the Al-xSi-3.5Fe alloys in 3.5 wt.% NaCl solution was tested, and the results showed that as the Si content increased, the electrode potential of the alloy first rose and then fell, the corrosion rate decreased initially and then increased, and the corrosion resistance first rose and then fell. When the Si content is 4 wt.%, the alloy has the strongest corrosion resistance; when the Si content is 10 wt.%, the alloy has the optimal activity. The research results show that the lower the corrosion potential, the higher the corrosion current density, the faster the corrosion rate, and the better the corresponding alloy activity.

Due to the higher electrode potential of the iron-rich phases (Al_6_Fe, Al_8_Fe_2_Si, and Al_9_Fe_2_Si_2_) and the eutectic Si phase compared to the aluminum matrix, galvanic corrosion occurs. Combined with the microstructure analysis of the corroded coating alloys, without adding Si elements, the iron-rich phase Al_3_Fe in the alloy mainly accumulates at the grain boundaries. Its electrode potential is higher, forming a significant potential difference with the aluminum alloy surface and activating the alloy to develop local sacrificial anode reactions—the electrode potential drops compared to pure Al. When the Si content is 1 wt.%, the iron-rich phase in the alloy transforms into the ternary alloy compound Al_8_Fe_2_Si and the eutectic phase Al-Al_8_Fe_2_Si, reducing the potential difference with the matrix and the decreasing galvanic corrosion, improving corrosion resistance and increasing the electrode potential. When the Si content is 2 wt.%, the iron-rich phase in the alloy does not change much. Still, the increase in Si content raises the electrode potential of the aluminum matrix, further reducing the potential difference between the iron-rich phase and the matrix. At the same time, the aggregation of iron-rich phases in the microstructure minimizes the tendency of aluminum alloys towards galvanic corrosion, which promotes electrode potential and improves corrosion resistance. When the Si content is 4 wt.%, the number of Al_8_Fe_2_Si phases significantly decreases, while Al_9_Fe_2_Si_2_ phases are generated along with a small amount of eutectic Si phase. The overall potential difference shrinks, galvanic corrosion slows down, and the electrode potential drops. When the Si content is 10 wt.%, a large number of Al_9_Fe_2_Si_2_ and eutectic Si phases are generated in the alloy, both of which act as cathode phases in the aluminum matrix, forming more corrosion micro-galvanic cells with greater potential differences, accelerating galvanic corrosion of the coating alloy and causing the electrode potential to drop.

The change trend in the corrosion rate measured after the full immersion experiment is consistent with the change trend in the corrosion rate calculated based on the corrosion current, indicating that the change in corrosion resistance of the alloy is the same as the electrochemical results. However, the corrosion rate measured after the full immersion experiment is lower than the corrosion rate calculated based on the corrosion current, because the immersion experiment is the result of long-term testing, and the corrosion products peeling off and the uneven surface structure of the alloy during the immersion process lead to different corrosion rates.

## 5. Conclusions

Al-xSi-3.5Fe alloys were analyzed using SEM, X-ray diffraction, and electrochemical experiments in this research. The effects of different Si contents on the microstructure and electrochemical properties of Al-xSi-3.5Fe alloys were studied. The conclusions are as follows:

1. α-Al and iron-rich phases exist in Al-xSi-3.5Fe coatings with different Si contents. As the Si content increases, the iron-rich phase transforms from Al_3_Fe, Al_6_Fe to Al_8_Fe_2_Si, and finally to Al_9_Fe_2_Si_2_. When the Si content exceeds 4 wt.%, the excess Si forms an eutectic silicon phase with the aluminum matrix.
2. The electrode potential of the alloy first rises and then falls with the increasing Si content, and the corrosion resistance follows the same trend. The sacrificial anode performance is the best when the Si content is 10 wt.%.
3. The corrosion mode of the Al-xSi-3.5Fe alloys is galvanic corrosion. When the Si content is below 4 wt.%, the iron-rich phase acts as the cathode phase and forms micro-galvanic cells with the aluminum matrix. When the Si content is above 4 wt.%, the eutectic silicon phase acts as the cathode phase, forming micro-galvanic cells with the aluminum matrix and iron-rich phase.
4. These findings are of great significance for optimizing the performance of the Al-xSi-3.5Fe alloy for hot-dip aluminum in industrial applications. The follow-up research can investigate the electrochemical performance of the alloy under different concentrations of Cl ions.

## Figures and Tables

**Table 1:** Composition of coating alloy (wt%).

Alloy Number	Al	Si	Fe
0Si	Bal.	0	3.5
1Si	Bal.	1	3.5
2Si	Bal.	2	3.5
4Si	Bal.	4	3.5
10Si	Bal.	10	3.5

**Table 2:** Corrosion Parameters of Al-xSi-3.5Fe Coating Alloys.

Alloy	E_corr_ (V_sce_)	I_corr_ (A/cm^2^)	B_a_ (mV/Decade)	B_c_ (mV/Decade)	U_corr_ (mm/y)
0Si	−821.11	2.1144 × 10^−7^	323.08	152.2	6.90
1Si	−798.02	1.2438 × 10^−7^	195.9	149.46	4.06
2Si	−778.37	8.6633 × 10^−7^	21.104	60.89	2.83
4Si	−741.11	3.7377 × 10^−8^	30.213	163.03	1.22
10Si	−834.36	2.4255 × 10^−7^	46.028	194.96	7.92

**Table 3:** EIS (Electrochemical Impedance Spectroscopy) Corrosion Parameters of Al-xSi-3.5Fe Coating Alloy.

Alloy	Rs (Ω·cm^2^)	Q		Rp (Ω·cm^2^)	Error (%)
		Y_o_ (Ω·cm^2^)	n		
0Si	7.590	1.851 × 10^−5^	0.8251	2.970 × 10^4^	3.79
1Si	12.69	1.024 × 10^−5^	0.8616	3.808 × 10^4^	5.14
2Si	10.42	8.130 × 10^−6^	0.8581	6.882 × 10^4^	4.72
4Si	8.931	1.238 × 10^−5^	0.8354	3.081 × 10^5^	4.60
10Si	7.742	8.827 × 10^−6^	0.9034	1.127 × 10^4^	3.58

**Table 4:** Full immersion corrosion results of Al-xSi-3.5Fe coating alloy.

Alloy	Before Corrosion (g)	After Corrosion (g)	V_corr_ (g/m^2^·h)
0Si	10.3651	10.3754	0.1341
1Si	10.5229	10.5325	0.1347
2Si	10.7775	10.7867	0.1278
4Si	10.6111	10.6181	0.0986
10Si	10.5905	10.6031	0.1750

## Data Availability

The raw/processed data required to reproduce these findings cannot be shared at this time due to legal or ethical reasons.
